# Targeted 5-HT_1F_ Therapies for Migraine

**DOI:** 10.1007/s13311-018-0615-6

**Published:** 2018-02-27

**Authors:** Marta Vila-Pueyo

**Affiliations:** Department of Basic & Clinical Neuroscience, Headache Group, James Black Center, King’s College London, 125 Coldharbour Lane, London, SE5 9NU UK

**Keywords:** Migraine treatment, 5-HT_1F_, Migraine, Lasmiditan, LY344864, LY334370

## Abstract

Migraine is a common neurological disease characterised by the presence of attacks of unilateral, severe head pain accompanied by other symptoms. Although it has been classified as the sixth most disabling disorder, the available therapeutic options to treat this condition have not progressed accordingly. The advance in the development of 5-HT_1_ receptor agonists for migraine, including 5-HT_1B/D_ and 5-HT_1F_ receptor agonists, has meant a major step forward towards the progression of a better treatment for migraine. Triptans have a limited efficacy, and their effect on vasoconstriction makes them unsafe for patients with cardiovascular and/or cerebrovascular diseases. Therefore, novel effective antimigraine treatments without cardiovascular effects are required, such as selective 5-HT_1F_ receptor agonists (ditans). Lasmiditan has much higher affinity for the 5-HT_1F_ receptor than for the vasoconstrictor 5-HT_1B_ receptor. This has been confirmed in preclinical studies performed to date, where lasmiditan showed no effect on vasoconstriction, and in clinical trials, where healthy individuals and patients did not report cardiac events due to treatment with lasmiditan, although it should be confirmed in larger cohorts. Lasmiditan crosses the blood-brain barrier and may act both centrally and peripherally on 5-HT_1F_ receptors expressed on trigeminal neurons. It is a well-tolerated compound that does not induce major adverse events. Although ongoing phase III clinical trials are needed to confirm its efficacy and safety, lasmiditan might offer an alternative to treat acute migraine with no associated cardiovascular risk. This review will focus on the characterisation of 5-HT_1_ receptor agonists and their effects as migraine therapies.

**Electronic supplementary material:**

The online version of this article (10.1007/s13311-018-0615-6) contains supplementary material, which is available to authorized users.

## Introduction

Migraine is a common neurological disease characterised by the presence of attacks of unilateral, throbbing head pain accompanied by sensitivity to movement and to visual, auditory and other inputs [1]. Although migraine has been classified as the sixth most disabling disorder and the first among neurological disorders [2], the available therapeutic options to treat this condition have not progressed accordingly. Nonetheless, recent advances in the understanding of migraine pathophysiology have allowed the development of new promising treatments, including the 5-HT_1F_ receptor agonists (ditans).

The focus of this review will be on the therapies based on targeting the 5-HT_1F_ receptor, with an approach of the role of 5-HT_1_ receptors in migraine, the results obtained through preclinical models, followed by a summary of the clinical trials performed to date.

## 5-HT_1_ Receptors and Migraine

The link between serotonin (5-HT (5-hydroxytryptamine)) and migraine pathophysiology dates from 1959 when Sicuteri discovered an increased urinary excretion of 5-HT metabolites during migraine attacks [3]. Since then, the interest in 5-HT receptors as migraine therapeutic targets deepened further when ergots, such as methysergide, came into use as a preventive medication. Further developments were made in the 1990s as therapies targeting the 5-HT_1_ receptor subselective agonists.

The classification of 5-HT receptors includes seven subfamilies, known as 5-HT_1_ to 5-HT_7_, and the subfamily most implicated in migraine is 5-HT_1_, which comprises five subtypes, known as 5-HT_1A, B, D, e_ and _F_ [4]. Table 1 summarises the main characteristics of these receptors.

**Table 1:** Summary of the main characteristics of 5-HT_1_ receptors

Receptor subtype	Distribution	Effector mechanism	Physiological action	Agonists used as antimigraine therapy
5-HT_1A_	CNS Raphe nuclei, hippocampus, amygdala, septum, entorhinal cortex, hypothalamus PNS Cholinergic heteroceptor in myenteric plexus	- Inhibition of adenylyl cyclase - Opening of K^+^ channels - Inhibition of voltage gated Ca^2+^ channels	- Serotonergic auto receptor - Neuronal inhibition - Facilitate ACh and NA release - Cholinergic nerve terminal in myenteric plexus - Hyperphagia	None
5-HT_1B_	CNS Subiculum, substantia nigra PNS Vascular smooth muscle	Inhibition of adenylyl cyclase	- Serotonergic auto receptor - Control release of ACh and NA - Contraction of vascular smooth muscle	Ergot alkaloids Triptans
5-HT_1D_	CNS Cranial blood vessel PNS Vascular smooth muscle	Inhibition of adenylyl cyclase	- Serotonergic auto receptor - GABAergic and cholinergic heteroreceptor - Vasoconstriction of intracranial blood vessel	Ergot alkaloids Triptans
5-HT_1e_	CNS Cortex striatum PNS mRNA in vascular tissue	Inhibition of adenylyl cyclase	Unknown	None
5-HT_1F_	CNS Cortex, spinal cord, hippocampus, locus coeruleus, hypothalamus, amygdala, cerebellum, dorsal raphe nucleus, pineal gland PNS Uterus, mesentery, vascular smooth muscle	Inhibition of adenylyl cyclase	Trigeminal neuroinhibition in guinea pig and rat	Lasmiditan

5-HT_1C_ was reclassified as 5-HT_2C_, thereafter it is not included in this table. Data taken from [4–7]

*ACh* acetylcholine, *CNS* central nervous system, *PNS* peripheral nervous system, *NA* noradrenaline

### 5-HT_1B_ and 5-HT_1D_ Receptors

5-HT_1B_ receptors are expressed in the CNS in both presynaptic and post synaptic locations [8]. They are mainly found as presynaptic inhibitory receptors in pain sensory neurons, in the basal ganglia and in the frontal cortex, where they act as terminal auto receptors [5]. They are also expressed outside the CNS on cerebral arteries and other vascular tissues [5].

The expression of 5-HT_1D_ receptors in the CNS is lower compared to 5-HT_1B_. This subtype is mainly found in cranial blood vessels and in vascular smooth muscle [5] and also in pain sensory neurons.

### 5-HT_1B/1D_ Receptor Agonists as a Treatment for Migraine

#### Ergot Alkaloids

The first specific antimigraine drugs that were developed are the ergot alkaloids. Ergot alkaloids consist of ergotamine and dihydroergotamine, used as acute treatments [9–11], and methysergide, used as preventive treatment [12–14]. All ergot alkaloids are non-specific 5-HT_1_ receptor agonists that also bind α-adrenoceptors and dopamine receptors [15]. Although the main action of ergot alkaloids is still unknown, initially, it was thought that their pharmacological effect of the greatest interest in migraine was arterial vasoconstriction through binding to 5-HT_1B_ receptors and α-adrenoceptors. However, they also exert a central effect through activation of 5-HT_1B_, 5-HT_1D_ and 5-HT_1F_ receptors on trigeminal nerve terminals, inhibiting in turn the release of vasoactive peptides preventing vasodilatation in migraine [16].

#### Triptans

Triptans were introduced as a migraine treatment in the 1990s, implicating a major step forward in the better treatment for this disorder [17]. Since then, new research has focused on new administration methods and formulations, including triptan combination therapies with NSAIDs [18, 19].

Triptans are selective 5-HT_1B/1D_ receptor agonists, although some have also affinity for the 5-HT_1F_ receptor [5], that lack many of the side effects induced by ergot alkaloids and that are selective to only migraine pain. Although the effect of triptans as antimigraine therapy is based on reducing neurotransmitter release from neurons mainly through the activation of 5-HT_1D_ receptors, their use is limited by their potential of blood vessel vasoconstriction through the activation of 5-HT_1B_ receptors which are found within the smooth muscle and in cerebral blood vessels endothelium. For this reason, the use of triptans is contraindicated in migraine patients with cardiovascular and/or cerebrovascular disease, uncontrolled hypertension and/or with particular forms of hemiplegic migraine [20]. Furthermore, their efficacy is limited as it has been described that in up to 25% of the patients none of the triptans is effective [21–23]. Of notice, the use of triptans on 10 or more days per month for at least 3 months has been linked to the development of medication overuse headache [24].

Triptans can be divided into two groups. One group includes almotriptan, eletriptan, rizatriptan, sumatriptan and zolmitriptan, which have faster onset and higher efficacy but have a higher propensity for recurrence. The other group includes frovatriptan and naratriptan, which have a better tolerability [25, 26].

## 5-HT_1F_ Receptor

The 5-HT_1F_ receptor gene was originally described in the mouse based on the homology of its sequence with the 5-HT_1B/1D_ receptors and was designated as 5-HT_1Eβ_ according to the similarity of pharmacology with 5-HT_1e_ receptor. Both subtypes, 5-HT_1e_ and 5-HT_1F_, have a high affinity for 5-HT but a low affinity for 5-carboxamidotryptamine (5-CT), which is a non-selective 5-HT_1_ agonist [27]. Table 2 summarises the binding affinity of 5-HT_1B/1D_ and 5-HT_1F_ receptor to several antimigraine compounds.

**Table 2:** Binding affinity (pKi) of 5-HT_1B,_ 5-HT_1D_ and 5-HT_1F_ to several compounds used as or developed to be antimigraine treatments

Compound	5-HT_1B_	5-HT_1D_	5-HT_1F_
Dihydroergotamine	9.2	9.4	6.6
Naratriptan	8.5	8.6	8.4
Rizatriptan	8	8.4	6.6
Sumatriptan	8	8.3	7.6
Zolmitriptan	8.3	9	7.6
LY334370	6.9	6.9	8.8
LY302148	7.3	7.7	8.6
LY306258	5.8	6.1	8.0
LY334864	6.3	6.2	8.2

The pKi value is for the human 5-HT_1B_, 5-HT_1D_ and 5-HT_1F_ receptors. For pKi values, affinity increases as the value increases. Adapted from [28, 29]

### Coupling of the 5-HT_1F_ Receptor

There are only a few studies that aim to identify the downstream signalling pathways of 5-HT_1F_ receptor. Their results have shown that 5-HT_1F_ receptors are negatively coupled to adenylyl cyclase in transfected cells, and some data indicates that the stimulation of these receptors can also induce phosphoinositide phospholipase C activation [30].

### Expression of the 5-HT_1F_ Receptor

In rodents, the 5-HT_1F_ receptor is widely expressed in the CNS, including in cerebral cortex [31, 32], periaqueductal grey [33], hippocampus [31, 34], trigeminal ganglia [35, 36], parafascicular and laterodorsal thalamic nucleus [32], dorsal root ganglia [35], amygdala [32], vestibular nuclei [37, 38], nucleus accumbens [32], putamen–caudate [31, 32], claustrum [31] and choroid plexus [31].

In humans, the mRNA of 5-HT_1F_ receptor is expressed in the CNS, thyroid and tonsils, kidneys, testis and ovaries [39]. Within the CNS, it is widely expressed, including in the main regions involved in migraine pathophysiology such as the cortex, the hypothalamus, the trigeminal ganglia, the locus coeruleus and the upper cervical cord [30, 32, 39–41]. It is also expressed in cerebral blood vessels such as the middle cerebral artery, although in lower concentrations and without vasoconstrictive properties [40, 42]. Its expression has been found very low in coronary arteries and absent in the heart [39, 42].

### Physiology of 5-HT_1F_ Receptor

5-HT_1F_ receptors are expressed in various locations of the trigeminovascular system, including peripherally in the trigeminal ganglion and centrally in the trigeminal nucleus caudalis (Sp5C). These receptors are located in both peripheral and central sensory trigeminal neurons, and their activation hyperpolarizes nerve terminals inhibiting trigeminal impulses [43, 44].

## 5-HT_1F_ Receptor Agonists for the Treatment of Migraine

### Development of 5-HT_1F_ Receptor Agonists for Migraine

Ergot alkaloids and triptans were developed under the theory that the pathophysiology of migraine headache was mainly attributed to the abnormal vasodilation of cranial blood vessels.

However, the results obtained throughout the past two decades of research have revoked this theory and have led to the conclusion that migraine is a neurological disorder and that cranial vasodilation may only be a secondary phenomenon induced by the activation of the trigeminovascular system [45–47]. In accordance to this, it should be noted that the action of ergot alkaloids and triptans is based on their effects on reducing neurotransmitter release from neurons mainly through the activation of 5-HT_1D_ receptors, but that their effects are limited by their action on vasoconstriction of cranial blood vessels through the activation of 5-HT_1B_ receptors.

The need to develop a selective treatment for migraine pain is crucial [48], especially when current specific treatment options, like triptans, have a limited efficacy [21–23] and are contraindicated in patients with cardiovascular disorders [20]. This need to develop a new antimigraine compound that lacks any cardiovascular and cerebrovascular effects increased the interest in studying the effects of 5-HT_1F_ receptor agonists as migraine treatments. Several selective 5-HT_1F_ receptor agonists have been developed in the past years, including lasmiditan (formerly known as LY573144 or COL-144), LY334370, LY344864 and LY349950. Only two of them, lasmiditan and LY334370, have been tested in human trials for migraine and have been proven efficient in attenuating migraine attacks. Unfortunately, the development of LY334370 as an antimigraine compound had to be stopped because of liver toxicity shown in dogs [44]. Thereby, this review will focus on lasmiditan as the unique 5-HT_1F_ receptor agonist currently in development as an antimigraine therapy.

### Lasmiditan

#### Properties of Lasmiditan

##### Chemistry of Lasmiditan

Lasmiditan is a novel 5-HT receptor agonist with high affinity and selectivity for the 5-HT_1F_ receptor. The chemical structure of lasmiditan is different from triptans, thereby is included in a novel drug class called “ditans”. The chemical difference between lasmiditan and triptans is based on the replacement of the indole structure of triptans, which is identical to the neurotransmitter 5-HT, by a pyridinoyl-piperidine scaffold [49].

##### Pharmacodynamics of Lasmiditan

Lasmiditan selectivity has been evaluated by using radioligand-binding techniques being compared against a panel of 50 receptors, ion channels and transporters. Of particular interest is the low-cross reactivity of lasmiditan with other members of the 5-HT_1_ receptor family showing a good selectivity based on binding affinity, with greater than 450-fold higher affinity at the 5-HT_1F_ receptor than at 5-HT_1A_, 5-HT_1B_ and 5-HT_1D_ receptors (Table 3). Furthermore, lasmiditan did not show significant affinity across a panel of monoaminergic receptor subtypes that regulate vascular tone (Table 4) [49].

**Table 3:** Binding affinity of lasmiditan at human 5-HT receptors

5-HT_1A_	5-HT_1B_	5-HT_1D_	5-HT_1F_	5-HT_2A_	5-HT_2B_	5-HT_2C_	5-HT_6_	5-HT_7_
1053 (± 134)	1043 (± 124)	1357 (± 156)	2.21 (± 0.22)	> 5 μM	> 2 μM	> 3 μM	> 4 μM	> 3 μM

Values are expressed as Ki in nanomolars (unless otherwise indicated) and represent the average ± SEM of more than five separate experiments. “>” indicates that less than 50% inhibition of binding was obtained at the specified concentration. For Ki values, affinity increases as the value decreases. Adapted from [49]

**Table 4:** Binding affinity of lasmiditan at other monoamine receptors

Adrenergic α_1_	Adrenergic α_2_	Adrenergic β_1_	Adrenergic β_2_	Dopaminergic D_1_	Dopaminergic D_2_	Histamine H_1_	Muscarinic
> 10	> 10	> 10	> 10	> 10	> 10	> 10	> 3

Values are expressed as Ki in micromolars and represent the average ± SEM of at least two separate experiments. “>” indicates that less than 50% inhibition of binding was obtained at the specified concentration. For Ki values, affinity increases as the value decreases. Adapted from [49]

Importantly, lasmiditan penetrates the blood-brain barrier and potentially could exert its effects centrally on the trigeminovascular system [50]. Moreover, the effects of lasmiditan could also be peripheral, acting on 5-HT_1F_ receptors expressed on trigeminal afferents or on the trigeminal ganglia. In both the central and the peripheral situations, lasmiditan may be dampening down the activation of second-order trigeminal neurons located in the trigeminal nucleus caudalis (Sp5C) which is the main mechanism currently believed to be involved in migraine pathophysiology [47].

Regarding the vasoconstrictor effects, in a rabbit saphenous vein model, lasmiditan showed no evidence of vasoconstriction, while comparable doses of sumatriptan induced 50% maximal vessel contraction [49]. The vascular effect of lasmiditan was also studied in dogs and in human isolated arteries and was compared to sumatriptan. In dogs, sumatriptan induced a statistically significant decrease in both coronary and carotid artery diameters. However, lasmiditan did not induce any vasoconstrictive activity at all doses tested [51]. In human isolated arteries, sumatriptan induced contractions in the proximal and distal coronary artery and in the internal mammary artery, in contrast with vehicle and lasmiditan that did not contract any of the arteries studied [51]. Table 5 summarises the effects of lasmiditan obtained in preclinical models.

**Table 5:** Summary of the effects of lasmiditan in preclinical models of migraine

Preclinical model	Results	Reference
Preclinical models of vasoconstriction		
Vasoconstriction model in rabbit saphenous vein	No vasoconstriction induced by lasmiditan while comparable doses of sumatriptan induced 50% maximal vessel contraction.	[49]
*In vivo* vascular constriction in dog	No vasoconstriction induced by lasmiditan at all doses tested, in contrast with sumatriptan that induced a statistically significant decrease in both coronary and carotid artery diameters.	[51]
Vasoconstriction model using human isolated arteries	Vehicle and lasmiditan that did not contract any of the arteries studied, in contrast with sumatriptan that induced contractions in the proximal and distal coronary artery and in the internal mammary artery.	[51]
Preclinical models of migraine pathophysiology		
*In* *vivo* dural plasma protein extravasation induced by electrical stimulation of trigeminal ganglion in rodent	Oral lasmiditan induced a concentration dependent decrease in the dural plasma protein extravasation after stimulation of the trigeminal ganglion.	[49]
*In* *vivo* activation of trigeminal second-order neurons in the TCC after electrical stimulation of trigeminal ganglion in rodent	Oral lasmiditan induced a concentration dependent reduction of neuronal activation in the TCC compared to placebo.	[49]
*In* *vivo* dural-evoked activation of trigeminal second-order neurons in the TCC in rodent	Intravenous lasmiditan significantly inhibited nociceptive dural-evoked neuronal activation in the trigeminal nucleus caudalis without having an effect on blood pressure.	[39]
*Ex vivo* analysis of CGRP release in rodent trigeminovascular system	Lasmiditan inhibited CGRP release from isolated preparations of dura mater, trigeminal ganglion and trigeminal nucleus caudalis.	[52]

*TCC* trigeminocervical complex

##### Pharmacokinetics of Lasmiditan

In rodents, 2 h after a single intravenous dose of lasmiditan (1 mg/kg), its concentration reached 249 and 161 nM in the brain and plasma, respectively, with a brain/plasma ratio of 1.57 and an unbound fraction of 50.5% of the brain [50].

The oral bioavailability of lasmiditan has been reported to be around 40%, and the time at which the maximum concentration of lasmiditan is found in serum (*T*_*max*_) ranges between 1.5 and 2.5 h after oral administration of 50–400 mg [53].

A placebo-controlled, randomised, dose-escalation study of lasmiditan showed dose linearity from 25 to 400 mg when given as an oral liquid in both females and males. The tablet achieved a comparable *C*_*max*_ and AUC to the liquid with only a slight delay in *T*_*max*_ [53].

#### Effects of Lasmiditan in Preclinical Models of Migraine

Preclinical models of migraine have been proven crucial to understand the specific mechanism by which treatments are effective in treating this disorder. At the same time, they are also a potent tool to discover new pathophysiological and molecular mechanisms involved in migraine.

Lasmiditan has been analysed in four different preclinical models of migraine in rodent. Although more studies are needed to properly understand the mechanisms by which lasmiditan is effective in treating migraine, the results obtained to date suggest an involvement of this compound in the modulation of the dural neurogenic inflammation and of the trigeminovascular system, which are key factors in the development of migraine. A detailed description of the results obtained is now explained and is also summarised in Table 5.

A model of dural extravasation in rodents showed that oral administration of lasmiditan induced a concentration dependent decrease in the dural plasma protein extravasation after stimulation of the trigeminal ganglion [49]. This model is based on the theory that migraine could result from a neurogenic inflammation of the dura mater that could be in turn initiated by an axon reflex of nociceptive nerve fibres [54]. However, this theory was considered outdated after as many as 10 compounds that blocked dural neurogenic inflammation in the preclinical model failed in migraine clinical trials [55].

In an *in vivo* model of electrical stimulation of the trigeminal ganglion in rodents, the activation of trigeminal second-order neurons located in the trigeminal nucleus caudalis (Sp5C), analysed by immunostaining of a neuronal marker of activation (cFos), was reduced 1 h after oral administration of lasmiditan [49]. This model is based on the theory that during migraine, the trigeminovascular system is disrupted and subsequently there is an increased neuronal activation in the trigeminal nucleus caudalis. Other antimigraine treatments, such as triptans, have been shown to inhibit the neuronal activation in the trigeminal nucleus caudalis; therefore, they have also been proven efficient in this model [56].

In a similar *in vivo* electrophysiology model of trigeminovascular activation, the effects of intravenous lasmiditan on dural-evoked trigeminovascular nociception were studied. The results indicated that lasmiditan significantly inhibits nociceptive dural-evoked neuronal activation in the trigeminal nucleus caudalis without having an effect on blood pressure [39]. This is a well-validated model that has been widely used to analyse the effects of antimigraine compounds in the trigeminovascular complex.

A recent study analysed the modulation of CGRP release by lasmiditan and sumatriptan in rodents [52]. Interestingly, the results indicated that both lasmiditan and sumatriptan significantly inhibited CGRP release from isolated preparations of dura mater, trigeminal ganglion and trigeminal nucleus caudalis, suggesting that the efficacy of these treatments may be due to this mechanism.

#### Effects of Other 5-HT_1F_ Receptor Agonists in Preclinical Models of Migraine

Since there are only four published studies analysing the effects of lasmiditan in preclinical models of migraine, we will extend the information including the results obtained with other 5-HT_1F_ receptor agonists. The analysis of these results can supplement the information obtained by using lasmiditan and therefore are useful to understand the possible mechanisms by which lasmiditan is effective as an antimigraine compound. A detailed description of the results obtained is now explained and is also summarised in Table 6.

**Table 6:** Summary of the effects of other 5-HT_1F_ receptor agonists in preclinical models of migraine

Preclinical model of migraine pathophysiology	5-HT_1F_ receptor agonist used	Results	Reference
*In vivo* dural plasma protein extravasation induced by electrical stimulation of TG in rodent	LY302148, LY306258, LY334370,naratriptan	All compounds inhibited dural plasma protein extravasation after intravenous administration, with a rank of potency: LY334370 > naratriptan > LY302148 > LY306258 = zolmitriptan > dihydroergotamine > sumatriptan > rizatriptan.	[28]
*In vivo* dural plasma protein extravasation induced by electrical stimulation of TG in rodent	LY344864	Oral and intravenous administration of LY344864 induced a significant inhibition of the dural plasma protein extravasation.	[57]
*In vivo* capsaicin-induced activation of trigeminal second-order neurons in the TCC in rodent	LY344864	Intraperitoneal injection significantly reduced cFos immunoreactivity in the TCC, indicating a decreased activation of trigeminovascular nociception.	[58]
*In vivo* capsaicin-induced activation of trigeminal second-order neurons in the TCC in rodent	LY344864	Sumatriptan and 5-HT_1F_ receptor agonist LY344864 decreased the capsaicin-induced neuronal activation in the trigeminal nucleus caudalis. LY344864 acted via 5-HT_1F_ receptors while sumatriptan acted via 5-HT_1B_ receptors.	[59]
*In vivo* dural-evoked activation of trigeminal second-order neurons in the TCC in rodent	LY334370	Intravenous administration of LY334370 produced a significant dose-dependent inhibition of the nociceptive dural-evoked neuronal activation in the trigeminal nucleus caudalis.	[60]
*In vivo* dural-evoked activation of trigeminal second-order neurons in the TCC in cat	LY344864, naratriptan	5-HT_1B_, 5-HT_1D_ and 5-HT_1F_ receptor agonists significantly inhibited the nociceptive vascular activity in trigeminal neurons with a rank order of activity 5-HT_1B_ > 5-HT_1D_ >> 5-HT_1F_.	[61]
*Ex vivo* analysis of CGRP release in rodent trigeminovascular system	LY344864	LY344864 inhibited CGRP release from isolated preparations of dura mater, but not from trigeminal ganglion and trigeminal nucleus caudalis.	[62]

*TCC* trigeminocervical complex, *TG* trigeminal ganglion

Different 5-HT_1_ receptor agonists, including the 5-HT_1F_ receptor agonists LY302148, LY306258 and LY334370, and the 5-HT_1B/1D_ receptor agonists dihydroergotamine, naratriptan, zolmitriptan and rizatriptan, were tested in a model of neurogenic dural inflammation in guinea pig. The results showed that all compounds inhibited dural plasma protein extravasation after intravenous administration, with a rank of potency: LY334370 > naratriptan > LY302148 > LY306258 = zolmitriptan > dihydroergotamine > sumatriptan > rizatriptan. The correlation of the intravenous ID_50_ values of these drugs in the neurogenic inflammation model with the affinity at each of the serotonin receptors indicated a significant correlation with affinity for the 5-HT_1F_ receptor [28]. These results indicate that 5-HT_1_ receptor agonists modulate the neurogenic dural inflammation and suggest that the 5-HT_1F_ receptor is the subtype that presents a higher involvement, which supports the results obtained with lasmiditan [49]. On the same year, a similar model of neurogenic dural inflammation was used in rat to test the effects of the 5-HT_1F_ receptor agonist LY344864. The researchers were able to replicate the results obtained previously in guinea pig, showing an inhibition of the dural protein extravasation after oral or intravenous administration of the compound [57].

In a model of intracisternal capsaicin-induced cFos immunoreactivity in the trigeminal nucleus caudalis in rodent, the i.p. injection of 5-HT_1F_ receptor agonist LY344864 significantly reduced the cFos immunoreactivity, indicating a decreased activation of trigeminovascular nociception [58]. These results suggest that 5-HT_1F_ receptor agonists modulate trigeminovascular nociception and support the results obtained with lasmiditan that showed a decreased nociceptive activation in the trigeminal nucleus caudalis.

A similar model where capsaicin was applied to the meningeal surface to induce cFos immunoreactivity in the trigeminal nucleus caudalis was used to compare the effects of sumatriptan and the 5-HT_1F_ receptor agonist LY344864. The results obtained indicated that both compounds decreased the capsaicin-induced neuronal activation in the trigeminal nucleus caudalis and that LY344864 acted through 5-HT_1F_ receptors while sumatriptan acted via 5-HT_1B_ receptors [59].

In another study, the 5-HT_1F_ receptor agonist LY334370 was used in vascular models, in pain preclinical models and in a migraine preclinical model to determine its specificity in migraine mechanisms [60]. The vascular models studied the effects of LY334370 on human arteries and on neurogenic dural vasodilation in rats. In none of the two models used LY334370 had an effect on vessel vasodilation. The pain models were used to study the effects of LY334370 on carrageenan-induced hyperalgesia in the rat and on nociceptive reflexes in the spinalized, decerebrate rabbit. In these cases, LY334370 had no significant effect. On the contrary, LY334370 induced a significant and dose-dependent inhibition of the nociceptive dural-evoked neuronal activation in the trigeminal nucleus caudalis. These results support the specificity of the 5-HT_1F_ receptor agonist in migraine against its vascular or general pain effects. Moreover, they are in line with the results obtained with lasmiditan.

A model of the dural-evoked activation of trigeminal nucleus caudalis in cat was used to compare the inhibitory effects of the activation of trigeminovascular nociception of several 5-HT_1B_, 5-HT_1D_ and 5-HT_1F_ receptor agonists, including naratriptan and LY344864 (both 5-HT_1F_ receptor agonists). The results indicated that the three subtypes of receptor agonists significantly inhibited the nociceptive trigeminovascular activation with a rank order of activity: 5-HT_1B_ > 5-HT_1D_ >> 5-HT_1F_ [61]. These results support the ones obtained in a similar model in rodent with lasmiditan [39].

Another study analysed the modulation of CGRP release by LY344864 in the isolated rat trigeminovascular system [62]. The results indicated that LY344864 was able to inhibit CGRP release in the dura mater, but not in the trigeminal nucleus caudalis or the trigeminal ganglia: in contrast with the results obtained in a similar model where lasmiditan inhibited CGRP release in the three structures [52].

#### Effects of 5-HT_1F_ Receptor Agonists in the Treatment of Migraine Attacks—Updated Summary of Results from Clinical Trials of Lasmiditan

We found 11 phase I, two phase II and three phase III clinical trials where lasmiditan was studied in different circumstances, doses and formulations. We only found the results of two of the phase II clinical trials published in journals; therefore, we completed the information using an official website for clinical trials [63] and a recent review [64].

##### Phase I Clinical Trials of Lasmiditan

The first clinical trial performed with lasmiditan dates from 2003. We could only find information about it in a recent review [64], where it was stated that 40 healthy individuals were administered intravenous lasmiditan to evaluate its safety, tolerability and pharmacokinetics. Five years later, two phase I clinical trials (COL MIG-102 and COL MIG-103) were performed in 44 individuals that were administered with either an oral solution or a sublingual tablet with the objective to assess bioavailability, safety, tolerability and pharmacokinetics [64]. We were not able to find the outcome of these trials published in papers. However, the results were briefly commented in a conference abstract where it was stated that all doses of solution were well tolerated without clinically significant effects on vital signs, orthostatic response or ECGS; at doses of 100 mg and above, the most common adverse signs were drowsiness, dizziness and paresthesia; doses of 50 mg and above achieved plasma levels that were previously associated with efficacy by the intravenous route and that the tablet achieved a similar *C*_*max*_ and AUC to the solution. The conclusion of these two trials was that lasmiditan is orally available and achieves plasma levels that were previously associated with efficacy after intravenous administration [65].

In 2011, the COL MIG-105 was performed confirming cardiac safety for lasmiditan, which was a major step forward for the development of the safety profile of lasmiditan. Specifically, lasmiditan did not cause QT prolongation in oral doses of 100 and 400 mg in healthy subjects in a comparator trial and did not cause arrhythmia or any pro-arrhythmic effects [23].

A randomised, open-label study was performed to compare the bioavailability of oral lasmiditan (200 mg) under fed and fasted conditions (COL MIG-104). The fed condition was associated with an increased *C*_*max*_, *T*_*max*_ and AUC and a lower rate of mild adverse effect (19 vs. 23) [66]. There were no serious adverse effects, and the most common side effect was somnolence [63, 64].

In 2017, two phase I clinical trials finished, although the results have not been released yet. One of the studies (COL MIG-110) involved the participation of eight healthy individuals that received single oral solution of [14C]-lasmiditan to test its absorption, metabolism and excretion [67]. The other study (COL MIG-118) involved the participation of 42 individuals in a randomised, double-blind, three period, cross-over study to evaluate single oral doses of lasmiditan (200 mg) when co-administered with single doses of sumatriptan (100 mg) [68].

There is one phase I clinical trial that is still ongoing but not recruiting more patients (COL MIG-106). It is a randomised, double-blind, placebo-controlled, five-period, cross-over study to assess the effects of a single oral tablet (100 or 200 mg) on simulated driving performance [69].

There are three phase I clinical trials that are currently recruiting individuals (COL MIG-113, COL MIG-114 and H8H-MC-LAHA). Two of them are multicentre, open-label studies with the aim to analyse the effects of a single oral dose of lasmiditan (200 mg) in subjects with normal or impaired renal function (COL MIG-113) [70] or with impaired hepatic function (COL MIG-114) [71]. The last phase I clinical trial will study the effect of age on the pharmacokinetics, safety and tolerability of oral lasmiditan (H8H-MC-LAHA) [72].

Details of phase I clinical trials of lasmiditan have been summarised in Table 7.

**Table 7:** Summary of the phase I clinical trials performed to date with lasmiditan

Study ID	Administration	Summary	Participants (received lasmiditan)	Status	Date started–finished	Reference
Phase I						
No ID	Intravenous infusion	Safety, tolerability and pharmacokinetics	55 (55)	Completed	2003	[64]
COL MIG-102	Oral solution and sublingual	Safety, tolerability and pharmacokinetics	44 (44)	Completed	2008	[64]
COL MIG-103	Oral solution and oral tablet	Bioavailability and pharmacokinetics	44 (44)	Completed	2008	[64]
COL MIG-104	Twice oral (200 mg each)	Randomised, open-label study to compare the bioavailability under fed and fasted conditions	30 (30)	Completed, with results	2014–2016	[64, 66]
COL MIG-105	Oral tablet	Thorough QT study	55 (55)	Completed	2011	[64]
COL MIG-106	Single oral tablet (100 or 200 mg)	Randomised, double-blind, placebo-controlled, five period, cross-over study to evaluate the effects on simulated driving performance	90	Ongoing, not recruiting	2016–2017	[69]
COL MIG-110	Single oral solution (200 mg)	Absorption, metabolism and excretion of [14C]-lasmiditan	8 (8)	Completed	2017	[67]
COL MIG-113	Single oral tablet (200 mg)	Multicentre, open-label, parallel-group adaptive pharmacokinetic single dose study in subjects with normal and impaired renal function	32*	Recruiting	2017	[70]
COL MIG-114	Single oral (200 mg)	Multicentre, open-label, parallel-group, pharmacokinetic single dose study in subjects with normal and impaired hepatic function	24*	Recruiting	2017	[71]
COL MIG-118	Single oral tablet (200 mg)	Randomised, double-blind, three period, cross-over study to evaluate single oral doses when co-administered with single doses of sumatriptan	42	Completed	2017	[68]
H8H-MC-LAHA	Single oral	Effect of age on the pharmacokinetics, safety and tolerability	36*	Recruiting	2017	[72]

*Indicates estimated enrolment

##### Phase II Clinical Trials of Lasmiditan

Two phase II clinical trials of lasmiditan have been performed to date, COL MIG-201 and COL MIG-202.

COL MIG-201 is a randomised, multicentre, placebo-controlled, double-blind, group-sequential, adaptive treatment-assignment, proof-of-concept and dose-finding study in the acute treatment of migraine that was performed during 2006 and 2007 [73–75]. It involved the screening of 372 subjects in 18 different centres in Finland, Germany and The Netherlands with 130 subjects being treated in-hospital during a migraine attack (42 received placebo and 88 lasmiditan). The doses used of intravenous lasmiditan ranged from 2.5 to 45 mg, and the 20 mg was identified as the effective dose that accomplished the primary outcome of the study, which was defined as “improvement from moderate or severe headache at baseline to mild or no headache at two hours post treatment”.

Lasmiditan was well tolerated, and there were no serious adverse events or clinically significant changes in vital signs, ECG parameters or in haematological/clinical chemistry parameters. The most frequently reported adverse events was paraesthesia which was reported to be mild and transient.

COL MIG-202 is a randomised, multicentre, placebo-controlled, double-blind, parallel-group, dose-ranging outpatient study in the acute treatment of migraine that was performed between 2009 and 2010 [76, 77]. It involved the screening of 534 patients in 43 European headache centres with migraine with and without aura who were not under prophylactic treatments for migraine, of whom 512 were randomly assigned to treat one moderate or severe attack at home with oral lasmiditan (50, 100, 200 or 400 mg) or placebo. The primary endpoint of the study was dose response for headache relief, defined as “improvement from moderate or severe to mild or none, at two hours post treatment”.

All lasmiditan doses significantly improved headache response at 2 h when compared to placebo with a significant linear association between headache response rate and lasmiditan dose and between headache free rate and lasmiditan dose. The highest dose of lasmiditan (400 mg) reduced headache severity at 30 min and, from 1.5 to 4 h post treatment, all doses of lasmiditan were superior to placebo. Interestingly, nausea, photophobia and phonophobia decreased in all treatment groups within 2 h after treatment, with the smallest decrease in the placebo group.

Lasmiditan was well tolerated, and there were no clinically significant changes in vital signs, ECG parameters or in haematological/clinical chemistry parameters. The most frequently reported adverse events were paraesthesia, dizziness or vertigo with an intensity ranging from mild to moderate. The results of this trial confirmed the results of the COL MIG-201 with the intravenous dosing, indicating that lasmiditan can dose-dependently improve acute migraine without vasoconstrictive activity when compared to placebo.

Details of phase II clinical trials of lasmiditan are summarised in Table 8.

**Table 8:** Summary of the phase II and phase III clinical trials performed to date with lasmiditan

Study ID	Administration	Summary	Participants (received lasmiditan)	Status	Date started–finished	Reference
Phase II						
COL MIG-201	Intravenous	Randomised, multicentre, placebo-controlled, double-blind, group-sequential, adaptive treatment-assignment, proof-of-concept and dose-finding study in the acute treatment of migraine	130 (88)	Completed	2006–2007	[63, 73–75]
COL MIG-202	Single oral (50, 100, 200 or 400 mg)	Randomised, multicentre, placebo-controlled, double-blind, parallel-group, dose-ranging outpatient study in the acute treatment of migraine	512 (86)	Completed	2009–2010	[63, 76, 77]
Phase III						
COL MIG-301 (SAMURAI)	Single oral tablet (100 or 200 mg)	Prospective, randomised, double-blind, placebo-controlled study in subjects with disabling migraine (Migraine Disability Assessment (MIDAS) score ≥ 11)	2231 (617)	Completed	2015–2016	[78]
COL MIG-302 (SPARTAN)	Single oral tablet (50, 100 or 200 mg)	Prospective, randomised, double-blind, placebo-controlled study in subjects with disabling migraine (MIDAS score ≥ 11)	3007	Ongoing, not recruiting	2016–2017	[79]
COL MIG-305 (GLADIATOR)	Single oral tablet with second dose for rescue (100 or 200 mg)	Prospective, randomised, open-label study in subjects with migraine who have completed COL MIG-301 or COL MIG-302 to evaluate the safety, tolerability and efficacy of long-term intermittent use of lasmiditan 100 mg and 200 mg as the first and second dose for the acute treatment of migraine	2580*	Recruiting	2015–2018*	[80]

*Indicates estimated enrolment or estimated finishing date

##### Phase III Clinical Trials of Lasmiditan

Three phase III clinical trials of lasmiditan have been started to date, COL MIG-301, COL MIG-302 and COL MIG-303.

COL MIG-301 (also known as SAMURAI) is a prospective, randomised, double-blind, placebo-controlled, outpatient study in subjects with disabling migraine, who have a migraine disability assessment (MIDAS) score higher than or equal to 11, that was performed in the USA between 2015 and 2016 [78, 81]. The results have not been published yet, but they were presented at the 5th European Headache and Migraine Trust International Congress in 2016 [82]. Two thousand two hundred thirty-one patients, with an average of over five migraine attacks per month and an average MIDAS score of 31, were randomly assigned to treat one migraine attack at home with oral lasmiditan (100 or 200 mg) or placebo. Over 25% of the patients included used prophylactic methods for migraine, and 82% of the patients had multiple cardiovascular risk factors or cardiovascular conditions.

The primary endpoint of the study was the efficacy of lasmiditan compared to placebo for headache relief at 2 h post treatment. The secondary endpoint was the efficacy of lasmiditan for freedom from some of the associated symptoms of migraine, including nausea, photophobia or phonophobia, 2 h after treatment. Patients indicated the presence or absence of the associated symptoms at the predose time point, identifying the “most bothersome” symptom.

Both lasmiditan doses (100 and 200 mg) were significantly more efficacious than placebo on headache relief at 2 h post treatment and were also significantly more effective than placebo in reducing migraine-related disability and improving patient global impression of change. Lasmiditan doses of 100 and 200 mg were also significantly more efficacious than placebo on the relief of the most bothersome migraine-associated symptom at 2 h post treatment. Lasmiditan was well tolerated, and there were no significant differences in cardiovascular adverse events in patients treated with lasmiditan vs placebo.

COL MIG-302 (also known as SPARTAN) is a prospective, randomised, double-blind, placebo-controlled, outpatient study in subjects with disabling migraine, who have a MIDAS score higher than or equal to 11, that was performed between 2016 and 2017 [79]. The study was expected to include up to 2226 migraine patients in 140 different centres in the USA, UK and Germany.

The primary endpoint of this trial is to evaluate the safety and efficacy of three doses of oral lasmiditan, 50, 100 and 200 mg, compared to placebo, at 2 h after dosing on freedom from migraine headache pain. The secondary endpoint is to evaluate the efficacy of the same doses of oral lasmiditan compared to placebo on freedom from the most bothersome associated symptom of migraine, including nausea, phonophobia or photophobia. The main difference between COL MIG-301 and COL MIG-302 is the inclusion of a lower dose of lasmiditan (50 mg) in the latest.

The results of SPARTAN were released very recently in an online communication [83]. Lasmiditan met both the primary and the secondary endpoints. Specifically, it was reported that 2 h after treatment with lasmiditan, the percentage of migraine pain-free patients was significantly greater compared to placebo in all three oral doses: 28.6% for 50 mg (*p* = 0.003), 31.4% for 100 mg (*p* < 0.001), 38.8% for 200 mg (*p* < 0.001) and 21.3% for placebo. Lasmiditan was also significantly effective in reducing the migraine-associated most bothersome symptom in patients 2 h after receiving their first dose: 40.8% for 50 mg (*p* = 0.009), 44.2% for 100 mg (*p* < 0.001), 48.7% for 200 mg (*p* < 0.001) and 33.5% for placebo. In line with previous studies, the most common adverse events after receiving lasmiditan were dizziness, paresthesia, somnolence, fatigue, nausea and lethargy.

COL MIG-303 (also known as GLADIATOR) is a prospective, randomised, open-label study in subjects with migraine who have completed COL MIG-301 or COL MIG-302 to evaluate the safety, tolerability and efficacy of long-term intermittent use of oral lasmiditan 100 and 200 mg for the acute treatment of migraine [80]. The trial is currently recruiting patients and is expected to enrol up to 2580 subjects who will be randomised to receive 100 or 200 mg of lasmiditan for up to eight migraine attacks per month for 1 year. The results of this trial will be used to build an appropriate safety database for lasmiditan, including long-term safety, tolerability and efficacy results. The estimated completion date is May 2018.

Details of phase III clinical trials of lasmiditan have been summarised in Table 8.

## Clinical Safety and Tolerability of Lasmiditan

Lasmiditan has been reported to be generally well tolerated with no serious adverse events. However, there is a need for extensive safety data coming from larger cohorts and from long-term studies: a need that will probably be covered with the results of the clinical trial COL MIG-303 (GLADIATOR).

The studies published to date report that oral and intravenous lasmiditan is well tolerated at doses ranging from 2.5 to 400 mg without inducing any major adverse events or clinically significant changes in vital signs, ECG parameters or in haematological or clinical chemistry parameters [23, 65, 73]. The most prominent and common adverse signs are dizziness and paraesthesia followed by drowsiness and somnolence [63, 73, 76], which are reported to be from mild to moderate in intensity and are more frequent when higher doses of lasmiditan are administered.

Lasmiditan does not cause arrhythmia or any pro-arrhythmic effects in healthy volunteers [23] and neither cardiac adverse events, chest pain or chest tightening were reported in migraine patients treated with lasmiditan [76].

Importantly, lasmiditan did not cause QT prolongation in oral doses of 100 and 400 mg in healthy individuals, whereas the antibiotic moxifloxacin led to QT prolongation. Patients did not report triptan-like chest symptoms in relation to the lasmiditan infusion [73].

Besides the clinical safety and tolerability of lasmiditan that make it a safe antimigraine treatment option, it is worth noting that the headache relief obtained at 2 h of receiving 20 mg intravenous (64% headache relief) or 400 mg oral (64% headache relief) lasmiditan is comparable to that obtained at 2 h of receiving 6 mg subcutaneous (69% headache relief) or 100 mg oral (61% headache relief) sumatriptan [21].

## Conclusions

The development of 5-HT_1_ receptor agonists, including 5-HT_1B/D_ and 5-HT_1F_ receptors, has meant a major step forward towards the progression of a better treatment for migraine. Triptans have a limited efficacy and their effect on vasoconstriction makes them unsafe for patients with cardiovascular and/or cerebrovascular diseases [17]. Consequently, novel effective antimigraine treatments without cardiovascular effects are required.

Lasmiditan has much higher affinity for the 5-HT_1F_ receptor than for the vasoconstrictor 5-HT_1B_ receptor [49]. This has been confirmed in preclinical studies performed to date, where lasmiditan showed no effect on vasoconstriction, and in clinical trials, where healthy individuals and patients did not report cardiac events due to treatment with lasmiditan, although it should be confirmed in larger cohorts. Lasmiditan crosses the blood-brain barrier and may act both centrally and peripherally on 5-HT_1F_ receptors expressed on trigeminal neurons, as it has been shown in preclinical studies [39]. It is a well-tolerated compound that does not induce major adverse events. Although ongoing phase III clinical trials are needed to confirm its efficacy and safety, lasmiditan might offer an alternative to treat acute migraine with no associated cardiovascular risk.

## Electronic Supplementary Materials

ESM 1 (PDF 498kb)
